# Price promotions offered by quick service restaurants in Australia: analysis from an obesity prevention perspective

**DOI:** 10.1017/S1368980021002688

**Published:** 2022-03

**Authors:** Evelyn SY Looi, Kathryn Backholer, Adrian J Cameron, Lily Grigsby-Duffy, Liliana Orellana, Gary Sacks

**Affiliations:** 1 Global Obesity Centre (GLOBE), Institute for Health Transformation, Deakin University, 221 Burwood Highway, Burwood, VIC 3125, Australia; 2 Biostatistics Unit, Faculty of Health, Deakin University, Burwood, VIC, Australia

**Keywords:** Fast food, Price promotion, Healthiness, Combination deals

## Abstract

**Objective:**

To assess the price promotions offered by major quick service restaurant (QSR) chains in Australia from an obesity prevention perspective.

**Design:**

Cross-sectional audit of ten of the largest QSR chains in Australia. We collected information regarding temporary price promotions and ‘combination deals’ offered by each chain over thirteen consecutive weeks in 2019–2020. We assessed the type of promotions, the magnitude of discount, and the energy content and healthiness of items promoted (based on Victorian Government criteria).

**Setting:**

Melbourne, Australia.

**Participants:**

Ten major QSR chains.

**Results:**

Temporary price promotions (*n* 196) and combination deals (*n* 537 on regular menus, *n* 36 on children’s menus) were observed across the ten selected QSR chains. In relation to temporary price promotions, the mean magnitude of discount for main menu items (*n* 75) was 41·7 %. The price reductions and energy content of combination deals varied substantially the by chain, the meal size and the sides/drinks selected as part of the ‘deal’. When the lowest-energy options (e.g. small chips, small sugar-free drink) were included as part of each combination deal, the mean energy content was 2935 kJ, compared to 5764 kJ when the highest-energy options (e.g. large fries, large sugar-sweetened drink) were included. Almost all available products were classified as unhealthy.

**Conclusion:**

Price promotions are ubiquitous in major QSR chains in Australia and provide incentives to consume high levels of energy. The action to restrict price promotions on unhealthy foods and ensure lower-energy default items as part of combination deals should be included as part of efforts to improve population diets and address obesity in Australia.

Unhealthy diets and excess body weight are leading contributors to the burden of disease in Australia and globally^(1)^. Australian adults and children are over-consuming discretionary (unhealthy) foods and beverages, with an average of 35 % of total daily energy coming from foods and beverages that are typically high in fats, free sugars and salt^(2)^. In 2017–2018, 25 % of children and 67 % of adults in Australia were classified as overweight or obese^(3,4)^. Unhealthy diets and obesity have substantial health, social and economic costs, with obesity alone estimated to cost Australia $8·6 billion each year^(5)^.

Meals prepared outside of the home have been increasingly identified as an important contributor to population diets^(6,7)^. In Australia, over the last three decades, meals prepared outside of the home have increased from 25 % to 34 % of household food expenditure^(8)^. Fast-food restaurants (defined as ‘restaurants that primarily provide consumers with largely pre-packed “quick” meals with little or no table service, and in which take-away orders are likely to account for a significant proportion of orders’^(9)^) represent a substantial proportion of out-of-home food purchases in Australia^(10)^. For example, in 2017, 44 % of the amount of money Australian households spent on eating out each week was reportedly spent in fast-food establishments^(10)^. In 2018, over 80 % of Australians aged over 14 years reported regularly consuming fast food^(11)^, with adolescents identified as the highest consumers^(7,12)^. Fast-food meals are typically both energy-dense and nutrient-poor^(13)^ and can contribute substantially to an individual’s daily energy intake^(14)^. A growing body of research indicates a positive association between fast-food consumption and BMI^(15)^ and other health outcomes, such as total cholesterol and type 2 diabetes^(12)^.

A range of factors has underpinned the relatively high contribution of fast food to Australian diets. These factors include high accessibility of fast-food restaurants (hereafter referred to as quick service restaurant (QSR) chains)^(16)^, highly pervasive and effective marketing practices of QSR chains^(17,18)^, and structural conditions that constrain time available for home cooking^(3,12,19)^. The pricing strategies of QSR chains, used in conjunction with other marketing tactics, are designed to increase sales and profitability through various means, including promoting new items, increasing frequency and size of orders, targeting and attracting different customer groups, and encouraging brand loyalty^(20)^. Price promotions used by QSR chains include everyday value pricing (promotion of consistent low-price menu items), temporary price promotions (discounts offered for a limited time), and bundling separate items together in a combination at a lower price than the total of the individual items (hereafter referred to as ‘combination deals’)^(21)^. Price promotions at QSR chains have been identified as having an influence on customers’ perceptions regarding the value of the offer and their purchase intentions^(22,23)^. There is also some evidence to show that price promotions influence consumer purchasing decisions in QSR chains^(21,24)^.

There have been only a limited number of studies that have investigated QSR price promotion strategies from a public health perspective^(18,25–27)^. A 2009 study conducted in Canada^(28)^ concluded that price promotions were effective in boosting fast-food sales by encouraging consumers to purchase more frequently or in larger quantities. Although lower-priced meals in this study were perceived by customers as better value for money, they were higher in energy density and lower in beneficial nutrients compared to higher-priced meals. In Australia, there has only been one small study, conducted in 2015, has examined pricing strategies of QSR chains with a health lens^(27)^. The study found that salads had the highest energy cost ($ per kJ) of products available. However, the study did not examine temporary price promotions or the magnitude of price discounts on offer. Accordingly, there is very limited available data on the prevalence and types of price promotion strategies of QSR chains. This study aimed to contribute to addressing this gap by assessing the price promotions (including temporary price promotions and combination deals) offered by major QSR chains in Australia from an obesity prevention perspective. The objectives of the study were to estimate the (a) prevalence, types and magnitude of price promotions offered by the major QSR chains and (b) energy content and healthiness of the products included as part of price promotions.

## Methods

This study used a cross-sectional design to audit food and beverage price promotions by major QSR chains in Melbourne, Australia. For the purpose of this study, a temporary price promotion refers to a reduction in price on a particular menu item or a group of menu items for a limited period of time^(29,30)^. Temporary price promotions are typically accompanied by communication of the promotion, which highlights the saving. In some cases, temporary price promotions indicate the time frame during which they apply, creating an expectation of time-limited availability^(29,30)^. Sometimes, but not always, temporary price promotions are given a unique promotion name (‘offer’), for example, ‘KFC’s Cheap as Chips’ or ‘Nando’s $11 WTF deal’. For this study, where a temporary price promotion included a group of menu items (e.g. a range of particular types of pizzas) as part of the same offer, we considered them as a single temporary price promotion.

A combination deal (also referred to as a ‘combo meal’) refers to several single-menu items offered together as a ‘bundle’, typically at a price lower than the sum of each individual item. In Australia, most combination deals comprised a main menu item (e.g. burger or fried chicken), and/or a side menu item (e.g. fries or side salad), and/or a drink.

### Sample selection

The twelve largest QSR chains in Australia, by market share, were identified from the Euromonitor Passport International database (Table 1). QSR chains were selected if they had an outlet in the Melbourne Central Business District, Victoria, and had nutrition and pricing information for their products available online. Ten of the twelve QSR chains satisfied the selection criteria: (1) McDonald’s, (2) KFC, (3) Pizza Hut, (4) Hungry Jack’s, (5) Subway, (6) Domino’s Pizza, (7) Red Rooster, (8) Oporto, (9) Nando’s and (10) Grill’d. Two QSR chains were excluded because one (Chicken Treat) did not have an outlet in Melbourne and another (Taco Bell) did not declare nutritional information or pricing on their website.

**Table 1:** Major QSR chains in Australia by market share, as of 2018

Parent company/corporate owner	QSR market share % (as of 2018)*	Chains (main type of food)
McDonald’s Corp.	29·1	McDonald’s (burgers)
Yum! Brands International Inc.	12·6	KFC (chicken) Pizza Hut (pizza) Taco Bell (chicken)
Restaurant Brands International Inc.	8·7	Hungry Jack’s (burgers)
Doctor’s Associates Inc.	6·5	Subway (sandwiches)
Domino’s Pizza Inc.	5·9	Domino’s Pizza (pizza)
Craveable Brands Ltd.	4·3	Red Rooster (chicken) Oporto (chicken) Chicken Treat (chicken)
Nando’s Group Holdings Ltd.	1·6	Nando’s (chicken)
Grill’d Pty. Ltd.	1·3	Grill’d (burgers)

* Based on the market share in Australia from the Euromonitor Passport International database (2019).

### Data collection

#### Temporary price promotions

All temporary price promotions from each chain were collected weekly for 13 weeks, between 25 November 2019 and 25 February 2020. Temporary price promotions were identified through three sources. Firstly, each QSR chain’s website was reviewed weekly to identify and record attributes of price promotions offered. We varied the day of the week that this data collection occurred across the study period. Secondly, if the QSR chain had a dedicated mobile application (app), we registered for an account to identify and record details of any additional promotions specific to the app. Finally, Frugal Feeds^(31)^, a third-party website that collates news and price promotion information of QSR chains through crowdsourcing, was checked each week to cross-check and supplement promotions identified through other sources. For each of the identified temporary price promotions, we recorded weekly in a pro forma spreadsheet: (1) name of promotion; (2) name of item(s) promoted; (3) nutrition information and portion size for all products involved in the promotion; (4) promoted price of each item; (5) regular price of each item; (6) date when the promotion was identified; (7) advertised start and end date of the promotion (where applicable); and (8) source where the price promotion was identified. This information was used to define a list of unique temporary price promotions offered by each chain. For each of the first 4 weeks of the data collection period, physical in-store visits (one store per chain, all located in the Melbourne Central Business District) were also conducted. This was to identify any discrepancies between the price promotions advertised online and in their physical chain outlets. No discrepancies were found over the 4-week period, after which physical in-store visits were ceased and price promotion data were collected through online sources only. The data from Frugal Feeds were found to directly match the data collected through the QSR chains’ respective websites and/or mobile apps.

#### Combination deals

Pricing data and nutrition information of all combination deals and related food items offered by each of the selected QSR chains was collected in February 2020 through their respective websites or apps. The combination deals were categorised according to whether they were part of the ‘regular menu’, ‘breakfast menu’ or ‘children’s menu’ (‘menu type’). Meal sizes were categorised based on the serving size of the included sides and/or drinks, as either ‘small’, ‘medium’, or ‘large’. These classifications were typically based on chain-specific serving sizes. Where the chain only specified two available sizes, the smallest one (commonly identified as ‘regular’) was classified as ‘medium’ and the largest one as ‘large’. The meal size of combination deals that were designed for sharing (e.g. included more than one ‘main meal’ item as part of the deal or were labelled as a ‘family’ size or ‘for sharing’) were classified as ‘shared’. If a default selection of sides and/or drinks was identified for each combination deal (i.e. the item was automatically included as part of the combination deal unless the consumer chose a different option from a pre-specified list), this was noted. Nutrition and portion size information of all relevant menu items were recorded in a pro forma spreadsheet.

### Data analysis

Product healthiness and energy contribution: the healthiness of products included in temporary price promotion offers and combination deals was assessed using the 2016 Victorian Department of Health and Human Services’ Healthy Choices Framework^(32)^. The framework was developed to classify the healthiness of food and beverages into three categories utilising a traffic light system based on the product type and detailed nutrition criteria: (1) ‘green’ indicates the ‘best choice’ (most healthy); (2) ‘amber’ indicates a less healthy option, where consumers are advised to ‘choose carefully’ and (3) ‘red’ denotes an unhealthy option, where consumers are advised to ‘limit their intake’. While the detailed criteria underpinning The Healthy Choices Framework are provided elsewhere^(32)^, some examples of the criteria are provided here to illustrate the way they were applied in this context. All deep-fried food items were classified as ‘red’, and any combination deals containing a deep-fried main or side item were classified as ‘red’. Single main items, such as sandwiches and wraps, with the energy of <= 1000 kJ/100 g were classified as ‘amber’^(32)^. Combination deals with energy content of <= 2500 kJ per serve without any deep-fried food items and with water selected as the drink option were also classified as ‘amber’. Fresh vegetables, fruits, water and coffee with no added sweeteners were classified as ‘green’. Salads containing a variety of vegetables and/or lean meat and/or reduced-fat cheese with either no or low-fat dressing were classified as ‘green’. Salads with regular-fat cheese and/or crumbed or coated meats and/or oil-based dressing were classified as ‘amber’. Salads that included deep-fried ingredients were classified as ‘red’. Where menu items included a choice regarding products or condiments included, the healthiest available options were used to classify the healthiness of the menu item. All classifications of the healthiness of menu items were conducted by two authors (EL and GS) independently. Where there were discrepancies in the initial classification between the two authors, differences were resolved by discussion, and all final classifications were mutually agreed.

For the purpose of assessing product contribution to average daily recommended energy intake, an average recommended daily energy intake of 8700 kJ was used for adults, in line with regulations for menu kilojoule labelling in Victoria^(33)^ and 7100 kJ for children. Recommended children’s energy intake was based on the average recommended daily energy intake using the reference values of an 8-year-old boy (7300 kJ) and girl (6900 kJ) who lead a lightly active lifestyle (physical activity level of 1·6)^(34)^.

#### Temporary price promotions

Unique temporary price promotion, that is, any temporary price promotion that was identified at least once in the 13-week period, was the unit of analysis. Where a unique temporary price promotion applied to a group of menu items (each with different nutritional values and/or regular prices), we calculated the mean energy content and mean price saving based on the particular menu items eligible to be included as part of the offer. Unique temporary price promotions were categorised into ‘product types’ based on whether the promoted product(s) were considered to be: (1) main meal(s) for one person; (2) combination deal(s) for one person (consisting of one main menu item and at least one side or drink, intended for consumption by one person); (3) combination deal(s) for sharing (consisting of more than one main menu item, or a very large main menu item, such as ‘family-sized’ pizza and whole chickens, intended for consumption by more than one person); (4) side(s) and/or drink(s) only; and (5) dessert(s). Data on unique temporary price promotions were summarised by promotion type and chain as: (1) total number; (2) mean price; (3) mean magnitude of price reduction (in $ and as a percentage of the regular menu price of the item(s) promoted); (4) mean energy content; (5) mean contribution to recommended daily energy intake; (6) mean energy cost (cents per 100 kJ) and (7) healthiness classification. As it was not always possible to determine the specific start and end date of each temporary price promotion (e.g. if the temporary price promotion had already begun when our data collection commenced, or if no definitive end date was advertised), we did not perform a detailed analysis of the duration of temporary price promotions observed.

#### Combination deals

Combination deals were counted as unique based on a combination of the ‘main meal’ item(s) and the size (e.g. small, medium and large) of the sides and/or drinks. For example, a ‘Small Big Mac ® meal’ and a ‘Large Big Mac ® meal’ from McDonald’s were each counted as separate combination deals. Each combination deal usually involved a range of sides and/or drinks available for selection. Therefore, we calculated the selection of items that would result in the lowest and highest values for (a) price savings and (b) energy content. Combination deals for each menu type (‘regular menu’, ‘breakfast menu’ and ‘children’s menu’) were reported by the QSR chain and by the meal size. Data on combination deals were summarised for the lowest and highest options as: (1) mean magnitude and percentage of price savings, calculated with reference to the cumulative price of the individual menu items included in the combination deal; (2) mean energy content; (3) mean contribution to average recommended daily energy intake; (4) number of combination deals that exceeded 30 % of average recommended daily energy intake; (5) mean energy cost (cents per 100 kJ); (6) mean incremental energy cost, calculated as the difference in the energy cost (cents per 100 kJ) of the combination deal and the primary main meal item (e.g. burger, fried chicken pieces) included as part of the combination deal, where applicable; and (7) healthiness classification. The metrics related to energy intake contribution and energy costs were not calculated for combination deals with a ‘meal size’ classified as ‘shared’.

Data for each of the outcomes of interest were summarised by QSR chain using means and 95 % CI. Where results are reported for all QSR chains combined, means and 95 % CI were estimated using linear mixed models with QSR chain as a random effect to account for clustering. All analyses were performed in Stata 16.1.

## Results

### Temporary price promotions

One hundred and ninety-six (*n* 196) temporary price promotions were identified during the 13-week period: 75 on ‘main menu items’, 25 on ‘combination deals for one person’, 54 on ‘combination deals for sharing’, 30 on ‘sides and/or drinks’, and 12 on ‘desserts’ (Table 2). Ninety-eight of the temporary price promotions applied to more than one menu item (average of three menu items per temporary price promotion).

**Table 2:** Unique temporary price promotions offered by ten of the major QSR chains in Australia between 25 November 2019 and 25 February 2020 (13 weeks)

Product type	Total number of unique temporary price promotions identified	Mean promotion price (AU $)	95 % CI	Mean price reduction of promoted menu item(s) relative to regular price (AU$)	95 % CI	Mean percentage (%) price reduction of promoted menu item(s) relative to regular price	95 % CI	Mean energy content (kJ) of promoted menu item(s)	95 % CI	Mean contribution of promoted menu item(s) to average daily recommended energy intake* (%)	95 % CI	Mean energy cost (cents per 100 kJ)	95 % CI	Classification as per Healthy Choices Framework†
Main meal items only	75	10·26	6·76, 13·75	7·31	5·25, 9·38	41·7	36·9, 46·4	5898	3446, 8350	67·8	39·6, 96·0	20	16, 23	97 % Red, 3 % Amber
Combination deals for one person	25	7·03	5·80, 8·26	5·68	3·88, 7·49	42·3	36·3, 49·1	3618	2698, 4539	41·6	31·0, 52·2	24	13, 34	96 % Red, 4 % Amber
Combination deals for sharing	54	23·74	19·15, 28·32	14·11	10·08, 18·13	37·0	28·5, 45·4	14 661	10 736, 18 586	NA‡		17	15, 18	100 % Red
Sides and/or drinks	30	4·51	3·33, 5·69	4·01	2·37, 5·66	47·7	42·4, 53·0	2974	1592, 4357	34·2	18·3, 50·1	18	12, 23	90 % Red, 10 % Green§
Desserts	12	8·92	1·66, 16·19	2·73	1·31, 4·15	36·8	15·9, 57·8	3964	1694, 6235	45·6	19·5, 71·7	18	11, 25	100 % Red

NA, not applicable.

Mean and 95 % CI estimated under a linear mixed model with chain as random effect.

* The average daily recommended energy intake for an adult is 8700 kJ.

† Classification based on the 2016 Victorian Department of Health and Human Services’ Healthy Choices Framework^(32)^.

‡ Not calculated due to the ‘sharing’ nature of the combination deals.

§ Only drinks without added sugar (e.g. coffee without added sugar).

Mean price reductions (as a proportion of regular prices) were similar across product types, ranging from 36·8 % for ‘desserts’ to 47·7 % for ‘sides and/or drinks’. Price-promoted menu items also had similar costs per kJ across product types, ranging from 17 cents per 100 kJ for ‘combination deals for sharing’ to 24 cents per 100 kJ for ‘combination deals for one person’. Price-promoted products, on average, contributed greater than a third of the average daily recommended energy intake for adults, with price-promoted ‘main meal items’ contributing 67·8 % on average and price-promoted desserts contributing 45·6 % on average. Almost all (97 %) of the temporary price-promoted items were classified as ‘red’ (unhealthy), with three price-promoted items classified as ‘green’ (healthy) on the provision that no sugar was added to price-promoted hot drinks (e.g. coffee without sugar), and three price-promoted items classified as ‘amber’ (less healthy option) as the price promoted sandwiches had the energy of less than 1000 kJ/100 g.

McDonald’s (*n* 51) offered the highest number of temporary price promotions during the period, followed by Hungry Jack’s (*n* 38), and Domino’s (*n* 34) (Table 3). Nando’s and Grill’d only had one temporary price promotion each during the study period. There was substantial variation with regard to promotion price and the magnitude of price savings across the selected QSR chains. Although Grill’d had only one price promotion, it offered the highest price reduction (as a proportion of regular prices) (50 %). This was followed by McDonald’s (48·4 %), KFC and Red Rooster (both 45·4 %). Pizza Hut and Subway offered the lowest percentage price reduction (19·6 % and 23·7 %, respectively). Subway was the only QSR chain that had all its price-promoted items classified as ‘amber’. Subway’s price-promoted items also had the lowest energy content (1558 kJ) on average, although the mean energy cost for price-promoted items was higher at Subway (45 cents per 100 kJ) compared to most of the other chains. A breakdown of the energy content of price-promoted items in the selected QSR chains can be found in Supplemental Table 1.

**Table 3:** Temporary price promotions offered by ten major QSR chains in Australia between 25 November 2019 and 25 February 2020 (13 weeks)

QSR chain	Total number of unique temporary price promotions identified	Mean promotion price (AU $)	95 % CI	Mean price reduction of promoted menu item(s) relative to regular price (AU$)	95 % CI	Mean percentage (%) price reduction of promoted menu item(s) relative to regular price	95 % CI	Mean energy content (kJ) of promoted menu item(s)	95 % CI	Mean contribution of promoted menu item(s) to average daily recommended energy intake* (%)	95 % CI	Mean energy cost (cents per 100 kJ)	95 % CI	Classification as per Healthy Choices Framework†
McDonald’s	51	5·02	3·88, 6·17	4·19	3·48, 4·91	48·4	44·5, 52·2	2668	2184, 3152	30·7	25·1, 36·2	17	15, 20	96 % Red, 4 % Green‡
Hungry Jack’s	38	8·77	6·91, 10·63	5·93	4·54, 7·33	41·2	38·9, 43·4	5155	4090, 6221	59·3	47·0, 71·5	18	16, 19	97 % Red, 3 % Green‡
Domino’s	34	12·08	8·75, 15·40	10·39	7·46, 13·31	43·0	38·6, 47·3	8560	6408, 10 712	98·4	73·7, 123·1	14	12, 17	100 % Red
Pizza Hut	23	29·03	25·41, 32·65	7·20	4·91, 9·48	19·6	13·5, 25·7	19 775	16 367, 23 183	227·3	188·1, 266·5	16	14, 19	100 % Red
KFC	22	12·54	8·72, 16·37	9·56	6·79, 12·33	45·4	39·6, 51·3	6816	4680, 8952	78·4	53·8, 102·9	18	16, 21	100 % Red
Red Rooster	21	15·18	10·68, 19·68	12·14	8·76, 15·52	45·4	39·0, 51·9	9342	6512, 12 172	107·4	74·9, 139·9	17	15, 20	100 % Red
Subway	3	6·98	4·44, 9·53	2·23	0·21, 4·25	23·7	14·0, 33·5	1558·17	1242, 1874	17·9	14·3, 21·6	45	22, 68	100 % Amber
Oporto	2	10·38	−3·92, 24·67	7·13	−19·88, 34·13	40·2	−85·0, 165·3	5090	1151, 9029	58·5	13·2, 103·8	21	−23, 65	100 % Red
Nando’s	1	11·00	--	4·85	--	30·6	--	4216	--	48·5	--	26	--	100 % Red
Grill’d	1	15·08	--	15·08	--	50·0	--	6827	--	78·5	--	22	--	100 % Red

QSR, quick service restaurant.

* The average daily recommended energy intake for an adult is 8700 kJ.

† Classification based on the 2016 Victorian Department of Health and Human Services’ Healthy Choices Framework^(32)^.

‡ Only drinks without added sugar (e.g. coffee without added sugar).

The duration of the temporary price promotions varied across chains. Some QSR chains did not include a start and end date to their promotions. Three QSR chains (Hungry Jack’s, Dominos and Pizza Hut) adopted a voucher system for their temporary price promotion campaigns, whereby a promotion ran for periods from 6 weeks to 4 months, with customers given a limited number of vouchers to use during that period. For some QSR chains, the same set of price promotions were observed to be repeated consecutively. For example, during the 13-week monitoring period, Hungry Jack’s had the same set of price promotions for three promotion cycles with each cycle running for 10 weeks through their voucher system.

### Combination deals – ‘regular menus’

On ‘regular menus’, a total of 537 combination deals were identified (Table 4). Hungry Jack’s offered the most combination deals (*n* 179), followed by McDonald’s (*n* 138) and KFC (*n* 103). Three QSR chains (Domino’s, Grill’d and Nando’s) did not offer combination deals on their regular menus. All the combination deals that included a drink had the option to select from a list of soft drinks, juices and/or water, with no default drink indicated. McDonald’s was the only QSR chain that provided a ‘green’ (healthy) salad as one of the available options for sides, while the other six QSR chains that offered combination deals only offered chips/fries as the side item. All combination meals on the ‘regular menus’ were classified as ‘red’ (unhealthy).

**Table 4:** Price savings, energy content and healthiness of combination deals available on ‘regular menus’ of selected major QSR chains in Australia (February 2020)

QSR chain	Meal size	Number of combination deals	Price saving per combination deal, relative to price of individual items								Energy content per combination deal								Classification as per Healthy Choices Framework†
			Mean reduction ($) – lowest-priced option	95 % CI	Mean reduction (% of price of individual items) – lowest-priced option	95 % CI	Mean reduction ($) – highest-priced option	95 % CI	Mean reduction (% of price of individual items) – highest-priced option	95 % CI	Mean energy content (kJ) – lowest-energy option	95 % CI	Mean contribution to average daily recommended energy intake1 – lowest-energy option (%)	95 % CI	Mean energy content (kJ) – highest-energy option	95 % CI	Mean contribution to average daily recommended energy intake* – highest-energy option (%)	95 % CI	
Hungry Jack’s (*n* 179)	Small	57	1·00	0·54, 1·46	8·2	5·2, 11·1	1·97	1·49, 2·45	15·3	12·5, 18·1	4214	3842, 4585	48·4	44·2, 52·7	4965	4586, 5343	57·1	52·7, 61·4	100 % Red
	Medium	57	2·66	2·16, 3·17	18·3	15·7, 20·9	3·12	2·61, 3·63	20·8	18·2, 23·4	4448	4074, 4821	51·1	46·8, 55·4	5406	5023, 5789	62·1	57·7, 66·5	100 % Red
	Large	57	2·70	2·18, 3·21	17·2	14·7, 19·8	2·70	2·18, 3·21	17·2	14·7, 19·8	4985	4620, 5350	57·3	53·1, 61·5	6335	5970, 6700	72·8	68·6, 77·0	100 % Red
	Shared‡	8	7·42	3·02, 11·82	30·9	24·3, 37·4	7·76	3·20, 12·31	31·8	25·3, 38·3	7359	4459, 10 260	NA§		8066	5013, 11 119	NA§		100 % Red
McDonald’s (*n* 138)	Small	46	1·59	1·43, 1·76	13·2	11·6, 14·8	4·29	4·13, 4·46	29·0	27·4, 30·7	2389	2138, 2640	27·5	24·6, 30·3	3693	3442, 3944	42·5	39·6, 45·3	100 % Red
	Medium	46	1·54	1·43, 1·65	12·0	11·0, 12·9	3·54	3·43, 3·65	23·9	22·8, 25·0	2389	2138, 2640	27·5	24·6, 30·3	4299	4049, 4550	49·4	46·5, 52·3	100 % Red
	Large	46	1·09	0·99, 1·20	8·3	7·4, 9·2	3·14	3·04, 3·25	20·8	19·8, 21·7	2389	2138, 2640	27·5	24·6, 30·3	4987	4736, 5238	57·3	54·4, 60·2	100 % Red
KFC (*n* 103)	Medium	49	1·63	1·34, 1·92	12·8	11·0, 14·5	5·19	4·57, 5·81	32·0	30·0, 33·9	2834	2594, 3074	32·6	29·8, 35·3	4828	4467, 5188	55·5	51·3, 59·6	100 % Red
	Large	48	0·45	0·01, 0·89	2·1	−1·3, 5·5	5·02	4·35, 5·69	26·8	24·7, 29·0	2854	2613, 3096	32·8	30·0, 35·6	6497	6133, 6862	74·7	70·5, 78·9	100 % Red
	Shared‡	6	13·46	6·72, 20·21	27·8	19·2, 36·4	22·40	14·36, 30·43	39·7	34·3, 45·2	14 315	9377, 19 254	NA§		23 387	14 572, 32 202	NA§		100 % Red
Subway (*n* 52)	Medium	30	1·55	1·55, 1·55	10·3	10·1, 10·6	1·55	1·55, 1·55	10·3	10·1, 10·6	3383	3097, 3669	38·9	35·6, 42·2	4447	4161, 4733	51·1	47·8, 54·4	100 % Red
	Large	22	1·55	1·55, 1·55	8·6	8·4, 8·8	1·55	1·55, 1·55	8·6	8·4, 8·8	4492	4093, 4890	51·6	47·1, 56·2	5556	5157, 5954	63·9	59·3, 68·4	100 % Red
Red Rooster (*n* 27)	Small	3	6·22	2·99, 9·45	45·4	35·7, 55·2	6·22	2·99, 9·45	45·4	35·7, 55·2	2844	1461, 4226	32·7	16·8, 48·6	329	1362, 5218	37·8	15·7, 60·0	100 % Red
	Medium	19	7·05	5·50, 8·61	35·0	30·5, 39·6	7·05	5·50, 8·61	35·0	30·5, 39·6	4066	3544, 4589	46·7	40·7, 52·7	4887	4311, 5462	56·2	49·6, 62·8	100 % Red
	Shared‡	5	8·58	4·52, 12·64	31·0	21·0, 41·1	8·58	4·52, 12·64	31·0	21·0, 41·1	6498	5267, 7730	NA§		7933	6040, 9825	NA§		100 % Red
Pizza Hut (*n* 22)	Medium	7	2·90	1·40, 4·40	23·2	13·9, 32·5	2·90	1·40, 4·40	23·2	13·9, 32·5	3366	2387, 4346	38·7	27·4, 50·0	4585	3565, 5606	52·7	41·0, 64·4	100 % Red
	Shared‡	15	−1·60	−3·75, 0·55	−6·2	−13·0, 0·6	11·40	7·32, 15·48	21·4	17·1, 25·7	17 191	13 525, 20 857	NA§		26 378	20 158, 32 599	NA§		100 % Red
Oporto (*n* 16)	Medium	12	3·88	3·28, 4·47	20·3	18·2, 22·3	4·01	3·25, 4·77	20·7	18·1, 23·4	4774	4316, 5232	54·9	49·6, 60·1	5713	5038, 6388	65·7	57·9, 73·4	100 % Red
	Shared‡	4	12·84	0·52, 26·20	25·4	3·8, 47·1	15·21	−2·66, 33·08	27·9	2·2, 53·6	12 598	8463, 16 732	NA§		17 662	4367, 30 958	NA§		100 % Red

QSR, quick service restaurant; NA, not applicable.

Domino’s, Grill’d and Nando’s did not offer combination deals.

* The average daily recommended energy intake for an adult is 8700 kJ.

† Classification based on the 2016 Victorian Department of Health and Human Services’ Healthy Choices Framework^(32)^.

‡ Shared refers to combination deals that were clearly designed for sharing (e.g. included more than one ‘main meal’ item as part of the deal or were labelled as a ‘family’ size or ‘for sharing’).

§ Not calculated due to the ‘sharing’ nature of the combination deals.

The magnitude of price savings on combination deals varied by chain and meal size, as well as by side or drink options selected (Table 4). When comparing medium-sized combination deals, based on the lowest-priced side and drink options, Red Rooster had the greatest average proportional price savings (35·0 %), followed by Pizza Hut (23·2 %) and Oporto (20·3 %). Pizza Hut’s sharing-sized combination deals were more expensive (6·2 % higher in price compared to the price of the individual items) if the lowest-priced menu items were selected as part of the combination deal. However, when selecting the highest-priced items in the combination deals, Pizza Hut offered a 21·4 % mean price saving.

**Fig. 1:**
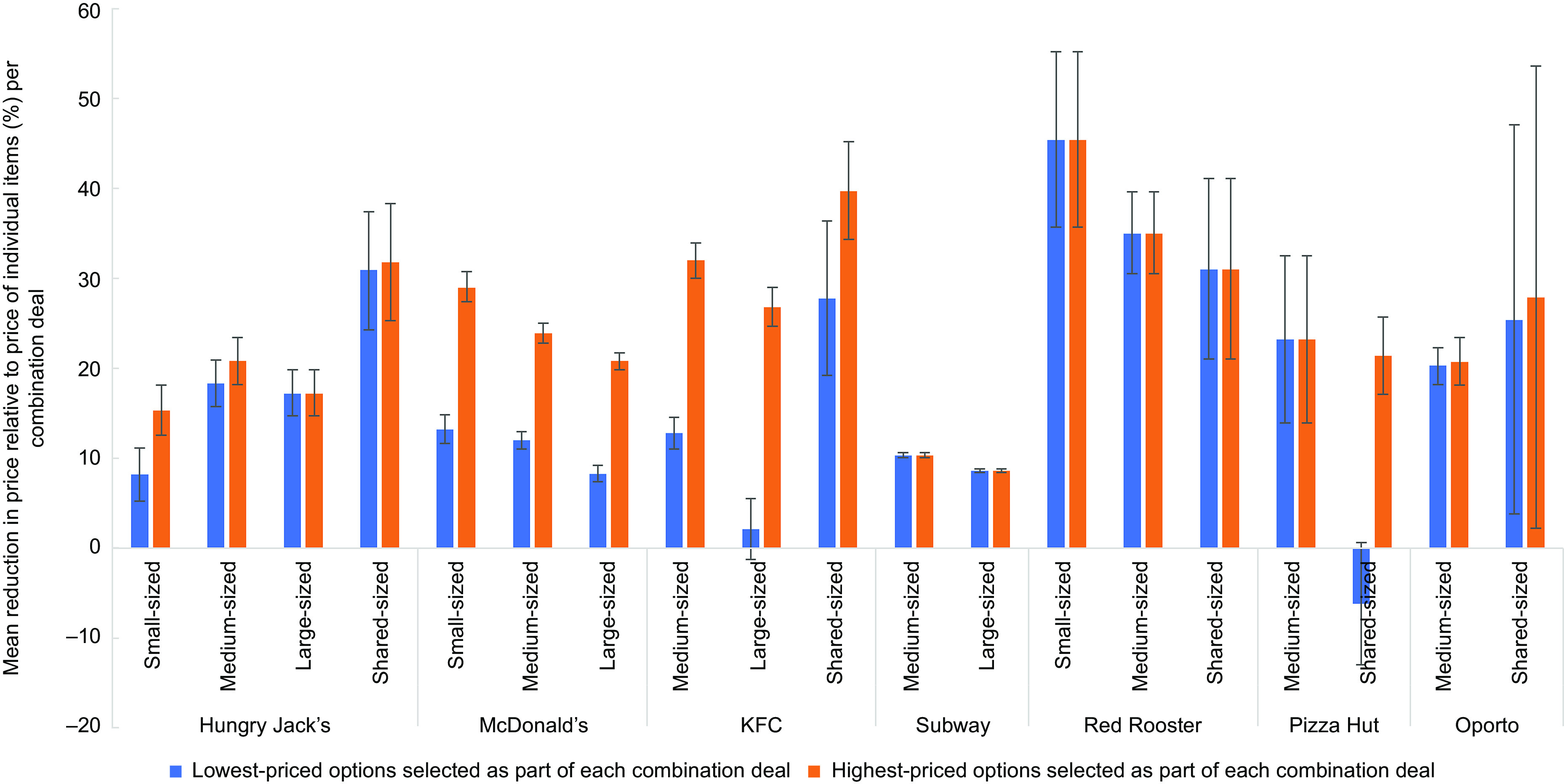
Mean percentage price reduction of combination deals (relative to the price of individual items) on ‘regular menus’ of selected major QSR chains in Australia, by meal size and product options selected as part of the combination deal, with 95 % CI (February 2020)

Across all of the selected QSR chains, the magnitude of price savings varied by the size of the combination deal (e.g. small, medium and large), with the small-sized combination deals often yielding greater savings. For example, the mean price reduction for McDonald’s small-sized combination deals with the lowest-priced side and drink options was 13·2 %, while the mean price reduction was 12·0 % and 8·3 % for their medium-sized and large-sized combination deals, respectively. Similarly, the mean price reduction for McDonald’s small-sized combination deals with the highest-priced side and drink options was 29 %, while the mean price reduction was 23·9 % and 20·8 % for their medium-sized and large-sized combination deals, respectively. The same pattern was observed for KFC, Subway and Red Rooster. For Hungry Jack’s and KFC, the value and proportion of the mean price savings for small-, medium- and large-sized combination meals was much lower than on shared combination meals, although this was not the case for Red Rooster, Pizza Hut and Oporto. Refer to Fig. 1.

**Fig. 2:**
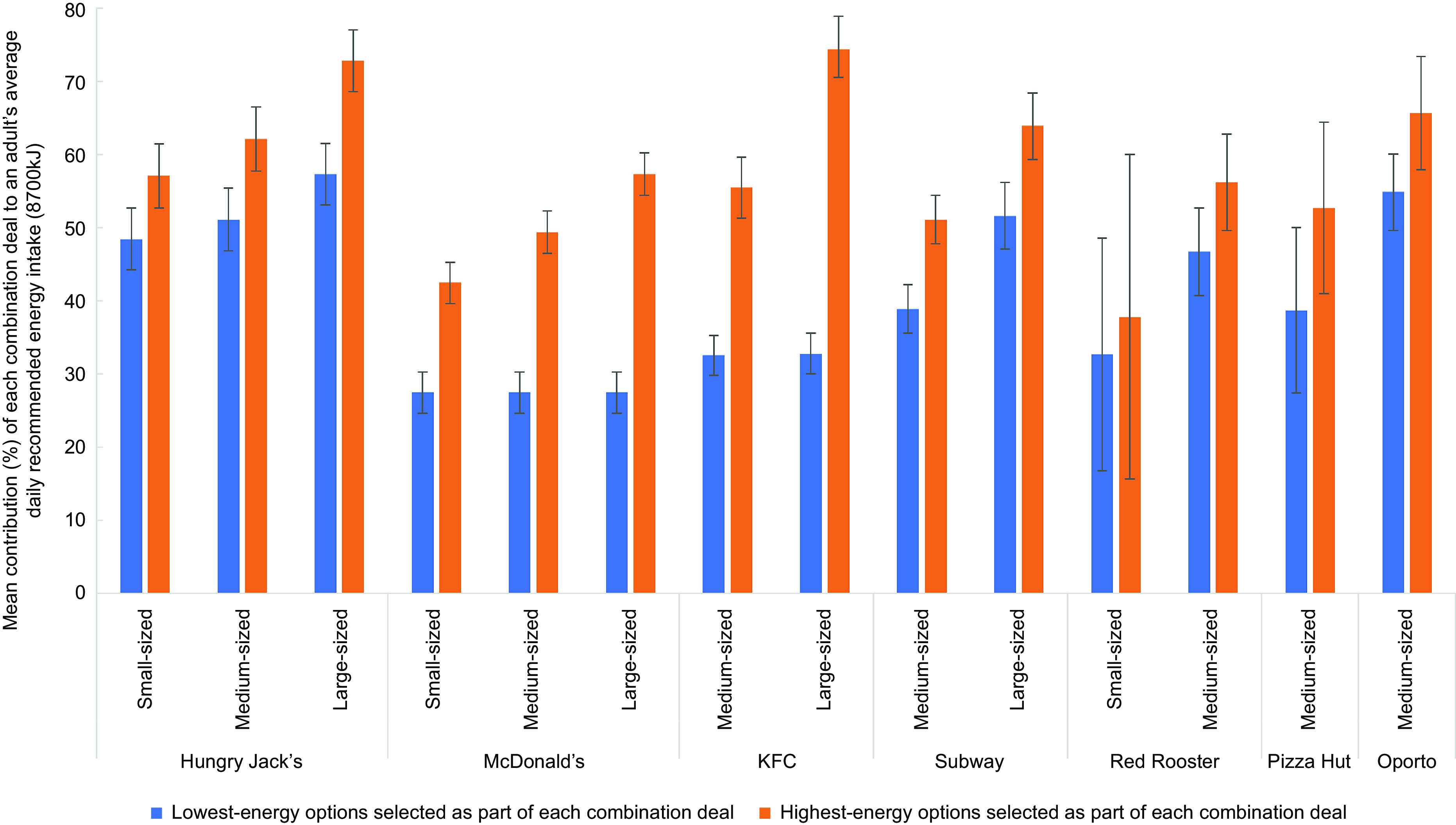
Mean percentage contribution of combination deals on ‘regular menus’ of selected major QSR chains in Australia to an adult’s average recommended daily energy intake (8700 kJ), by meal size and product options selected as part of the combination deal, with 95 % CI (February 2020)

With regard to energy content, 69 % of combination deals (excluding those with a ‘meal size’ classified as ‘shared’) provided greater that 30 % of an adult’s average recommended daily energy intake (equivalent to 2610 kJ) if the lowest-energy options were selected (see online Supplemental Table 2). This increased to 99 % of combination deals if the highest-energy options were selected. When comparing small-sized combination deals, Hungry Jack’s had the highest average energy content, with their small-sized combination deal contributing almost half (48·4 %) of an adult’s average recommended daily energy intake if the lowest-energy options were selected, and more than half (57·1 %) of an adult’s average daily recommended energy intake if the highest-energy options were selected (Table 4). At McDonald’s, if the low-kilojoule single-sized salad option was selected as part of their combination deals, with water or other sugar-free drink, they offered the lowest energy content combination meals on average, contributing to 27·5 % of an adult’s average daily recommended energy intake regardless of the size of the combination deal. However, if the highest-energy sides and drinks were selected at McDonald’s, the mean contribution of combination deals to average daily recommended energy intake would increase to 42·5 % for small-sized meals, 49·4 % for medium-sized meals and 57·3 % for large-sized meals. Large-sized combination deals with the highest-energy sides and drinks contributed substantially to average daily recommended energy intake at Hungry Jack’s (mean = 72·8 %) and KFC (mean = 74·7 %). Refer to Fig. 2.

In considering energy cost (price per kJ), we found that combination deals provided substantially cheaper energy compared with relevant stand-alone main meal items. However, this was only the case when the highest-energy sides and drinks (e.g. chips and sugary drink) were selected, not when lowest-energy sides and drinks (e.g. salad and/or water or sugar-free beverages) were chosen (see online Supplemental Table 3).

### Combination deals – ‘children’s menus’

A total of 34 combination deals, from eight of the ten QSR chains, were identified on ‘children’s menus’ (refer to Table 5). McDonald’s had the most combination deals on their children’s menu (*n* 7), while KFC had the fewest (*n* 2). All but one (KFC) of the eight QSR chains offered only one size of combination deal on their children’s menu.

**Table 5:** Price savings, energy content and healthiness of combination deals available on children’s menus of selected Australian QSR chains (February 2020)

QSR chain*	Number of meals	Price saving per combination deal, relative to price of individual items								Energy content per combination deal								Classification as per Healthy Choices Framework‡
		Mean reduction ($) – lowest-priced option	95 % CI	Mean reduction (% of price of individual items) – lowest-priced option	95 % CI	Mean reduction ($) – highest-priced option	95 % CI	Mean reduction (% of price of individual items) – highest-priced option	95 % CI	Mean energy content (kJ) – lowest-energy option	95 % CI	Mean contribution to average daily recommended energy intake† – lowest-energy option (%)	95 % CI	Mean energy content (kJ) – highest-energy option	95 % CI	Mean contribution to average daily recommended energy intake† – highest-energy option (%)	95 % CI	
McDonald’s	7	0·75	−0·13, 1·63	9·4	−5·97, 24·6	5·45	4·57, 6·33	47·5	42·5, 52·4	974	705, 1244	13·7	9·9, 17·5	3297	2876, 3718	46·4	40·5, 52·4	71·4 % Red, 28·6 % Amber
Grill’d	6	4·50	0	26·5	0	4·50	0	26·5	0	2553	1070, 4037	36·0	15·1, 56·9	2553	1070, 4037	36·0	15·1, 56·9	100 % Red
Hungry Jack’s	4	1·26	0·29, 2·24	17·1	4·0, 30·2	2·21	1·24, 3·19	26·7	15·2, 38·1	1214	725, 1702	17·1	10·2, 24·0	2911	2422, 3399	41·0	34·1, 47·9	100 % Red
Red Rooster	4	4·24	−0·20, 8·67	40·01	11·4, 68·8	6·18	1·74, 10·61	50·0	30·2, 69·8	1275	548, 2002	18·0	7·7, 28·2	2225	1498, 2952	31·3	21·1, 41·6	75 % Red, 25 % Amber
Subway§	4	NA		NA		NA		NA		948	889, 1007	13·4	12·5, 14·2	948	889, 1007	13·4	12·5, 14·2	100 % Amber¶
Oporto	4	2·68	0·98, 4·38	23·49	12·7, 34·3	2·68	0·98, 4·38	23·5	12·7, 34·3	2500	1903, 3097	35·2	26·8, 43·6	2500	1903, 3097	35·2	26·8, 43·6	100 % Red
Nando’s§	3	NA		NA		NA		NA		1337	597, 2076	18·8	8·4, 29·2	1837	409, 3264	25·9	5·8, 46·0	100 % Red
KFC‖	2	2·08	−10·31, 14·46	28·15	−98·6, 154·9	4·88	−7·51, 17·26	49·1	−15·0, 113·3	1202	598, 1805	16·9	8·4, 25·4	2895	2291, 3498	40·8	32·3, 49·3	100 % Red
Total	34	2·20	1·43, 2·96	21·3	14·9, 27·8	4·26	3·45, 5·08	37·5	31·5, 43·4	1538	1225, 1851	21 7	17·3, 26·1	2472	2139, 2804	34·8	30·1, 39·50	80·8 % Red, 19·8 % Amber

QSR, quick service restaurant; NA, not applicable.

* Pizza Hut and Domino’s did not offer any children’s combination deals.

† Based on an average recommended daily energy intake of 7100 kJ. This value is the average of the recommended daily energy intake reference value of an 8-year-old boy (7300 kJ) and girl (6900 kJ) who lead a lightly active lifestyle (physical activity level of 1·6)^(34)^.

‡ Classification of combination deals based on the 2016 Victorian Department of Health and Human Services’ Healthy Choices Framework^(32)^.

§ These QSR chains did not offer the main meal items in the children’s combination deals as individual or ala carte items; hence, the price savings could not be calculated as individual pricing for the main meal items were unavailable.

‖ KFC offered a large-sized for their two children’s combination deals. For this analysis, the ‘large-sized’ versions of these combination deals were excluded from analysis, as KFC was the only QSR chain that offered a large size.

¶ When default side (fruit purée) and drink (bottled water) options are selected.

Across the selected QSR chains, the mean percentage of price savings relative to the price of individual items included in children’s combination deals were 21·3 % for the lowest-priced options and 37·5 % for the highest-priced options. The mean energy content of children’s combination deals was 1538 kJ for the lowest-energy options and 2472 kJ for the highest-energy options. The mean contribution of combination deals to the average recommended daily energy content of an 8-year-old child was 21·7 % if the lowest-energy option of the side and drink were selected. However, this rose to a mean contribution of 34·8 % if the highest-energy option of the side and drink were selected, with combination deals from several chains, including McDonald’s, Hungry Jack’s and KFC, contributing over 40 %. 56 % of combination deals contributed greater than 30 % of an 8-year-old child’s average recommended daily energy intake (equivalent to 2130 kJ) if the highest-energy options were selected (see online Supplemental Table 4).

As was the case in relation to ‘regular menus’ and ‘breakfast menus’ (see online Supplemental Table 5), energy cost (price per kJ) decreased substantially with the purchase of a combination deal (compared with relevant stand-alone main meal items) when the highest-energy sides and drinks (e.g. chips and sugary drink) were selected. However, this was not the case not when lowest-energy sides and drinks (e.g. salad and/or water or sugar-free beverages) were selected (see online Supplemental Table 6).

Overall, 80·8 % of the children’s combination deals were classified as ‘red’ (unhealthy). All of Subway’s children’s combination deals were classified as ‘amber’ when the lowest-energy sides, drinks and condiments were selected. However, they would be classified as ‘red’ if different selections were made. When the lowest-energy options were selected, McDonald’s had 28·6 % of their children’s combination meals classified as ‘amber’, while Red Rooster had 25 % of their combination meals classified as ‘amber’.

## Discussion

In this audit of ten of the largest QSR chains in Melbourne, Australia, we found extensive use of price promotions. All of the included QSR chains offered a wide range of temporary price promotions and used ‘combination deals’ to offer discounts to consumers if they bought multiple items at one time. QSR chains offered a discount of approximately one-third to a half off the original prices of individual food and beverage items. With regard to combination deals, the energy content, energy cost and price discount (compared to the sum of the price of the individual items) varied substantially based on the meal size and the particular sides and drinks selected. The majority of the large-sized combination deals contributed more than half of an adult’s average recommended daily energy intake. All of the combination meals on regular menus were classified as ‘red’ (unhealthy) according to the Healthy Choices Framework.

When considering all combination deals and temporary price promotions offered by the QSR chains included in this study, our findings are in line with the findings of previous research^(18,27)^ that combination deals with higher energy content sides and drinks options yielded lower energy costs. Additionally, our results indicate that individuals would consume a substantial amount of energy if sides and drink options with higher energy content (e.g. chips and sugar-sweetened drinks) were selected as part of combination deals. Most of the QSR chains had high-energy chips/fries as the only available option as part of combination deals. This finding is in line with a 2019 study^(35)^ conducted in the UK that highlighted the high energy content of the sides and desserts sold in QSR chains.

In relation to children’s menus, most QSR chains included in this study provided high-energy side and drink options in their children’s combination deals, with substantial price savings (ranging between 21·3 % and 37·5 % depending on the side and drink selection) relative to stand-alone meal items. And 80·8 % of combination meals on children’s menus were classified as ‘unhealthy’. Overall, the children’s combination deals contribute between 21·7 % and 34·8 % of an 8-year-old child’s average recommended daily energy intake of 7100 kJ; however, this value was over 40 % for the highest-energy options for a number of QSR chains. These results are similar to previous observations for Australian QSR chains^(36)^. The findings are also in line with a study conducted in Guatemala^(17)^, where the authors also found that QSR chains utilised price incentives on children’s combination deals, which were all classified as ‘less healthy’.

The price promotions offered on unhealthy children’s meals occurred despite industry commitments to reduce marketing of unhealthy foods to children. The Australian Food and Grocery Council (AFGC) introduced a voluntary Quick Service Restaurant Initiative for Responsible Advertising and Marketing to Children (QSRI)^(37)^ in 2009 (updated in 2014), which set out a common framework purportedly to ensure that healthier choices were being promoted by signatory QSR chains. Six QSR chains, including McDonald’s, Hungry Jack’s, KFC, Oporto, Red Rooster and Pizza Hut (under the parent company, Yum! Restaurants International) signed up for the QSRI. Despite McDonald’s pledge of restricting marketing and advertising to children less than 14 years old in a range of settings, as outlined in their QSRI Action Plan^(38)^, they offered a number of children’s combination deals (referred to by McDonald’s as ‘Happy Meals’) targeted at children below 14 years of age. Almost three-quarters (71·4 %) of McDonald’s children’s combination deals were classified as ‘red’ (unhealthy). Similarly, Oporto made a commitment regarding the energy content of their children’s combination deals in their company action plan^(39)^ outlining that they would refrain from offering combination deals that target children between 4 and 8 years old with energy content of above 2080 kJ per meal. At the point that we conducted this study, we found that the lowest average energy content of children’s combination deals offered by Oporto was 2500 kJ. Previous research, conducted in 2018^(40)^, found that Subway had a commitment to providing a healthier side and drink option as the default option in their children’s combination deals. Our results indicated that they are adhering to this commitment, with all children’s combination deals offered by Subway classified as ‘amber’ (when the default sides and drinks were selected).

### Implications of findings

Price has a crucial influence on food choices^(27,41–43)^. The pervasiveness of price promotions and bundling incentives on energy-dense fast-food items is concerning from a public health perspective as current pricing strategies provide incentives to consume high levels of energy. Accordingly, the pricing strategies are likely to contribute to unhealthy diets and obesity. As such, routine monitoring of prices and price promotions in the fast-food sector, and their impact on consumer purchases and consumption, needs to be an important component of strategies to improve population diets and address obesity.

While some QSR chains in Australia have made some commitments to restrict marketing to children and improve the healthiness of products directed at children, these commitments are very limited and do not include price promotions. Moreover, there is consistent evidence that food industry self-regulation in regard to nutrition has largely proven to be ineffective^(44–46)^. As a result, governments should consider implementing policies that restrict price promotions of unhealthy fast food. While we are not aware of any governments that have taken action in relation to price promotions on fast food, recent policy announcements from the United Kingdom related to restrictions on multi-buy promotions for packaged foods high in salt, fat or sugar provide an example of the type of policies that could be implemented^(47)^.

For QSR chains willing to take action to address unhealthy population diets, they could consider providing healthier options (e.g. low-energy salads, grilled vegetables) as part of the combination deals they offer and making them the default option (i.e. healthier sides and drinks are automatically included as part of combination deals unless the consumer chooses a different option from a pre-specified list). For example, McDonald’s could have their garden salad and water as the default side and drink options as part of its combination deals, with customers still provided a range of other options from which to choose. A switch to healthier sides and drinks as the default options echoes the recommendations provided in a recent assessment of the nutrition-related policies of major QSR chains in Australia^(48)^. In addition, QSR chains could adjust their pricing of combination deals to ensure that there are no pricing incentives to purchase larger sizes. This may encourage customers to choose smaller-sized meals. From a public health perspective, there is evidence that policies that change the environment in which individuals make choices, such as recipe reformulation and default healthy drinks with children’s meals, tend to be more effective and equitable than interventions that rely exclusively on an individual to make healthier choices^(49–51)^. This is supported by the observation that, despite salads being made available for sale at McDonald’s, they represented a very small proportion of sales^(52)^. Moreover, interventions that do not directly restrict individual choice are likely to be acceptable to a range of stakeholders^(53)^.

### Strengths and limitations

This is the first study in Australia to comprehensively assess the temporary price promotions and pricing of combination deals offered by major QSR chains from an obesity prevention perspective. The study only examined temporary price promotions and combination deals on offer over a relatively short period (13 weeks). Our review of QSR chain websites for temporary price promotions was conducted weekly, and so it is possible that we may have missed price promotions that were only on offer for durations of shorter than a week. However, such promotions are likely to have been identified through our use of other relevant data sources (QSR chain apps and a third-party website). We classified temporary price promotions that applied to a group of menu items as a single instance of a price promotion, and so our identified number of price promotions observed can be viewed as conservative. While the study results are likely to be indicative of the types of price promotions offered by these QSR chains in Australia, future studies should examine price promotions over a longer period and for a broader set of QSR chains to understand variations by time of year and across different companies. Repeated studies would be valuable in understanding changes over time. While we were able to add nuance to our assessment of the energy content and healthiness of menu items by reporting a range of values for menu items that consisted of a range of sizes and options, it nevertheless proved challenging to assess and summarise the healthiness of some menu items that are highly customisable and/or designed for sharing. The highly customisable nature of QSR menu items thus needs to be taken into account when comparisons by QSR chain are made. In addition, more fine-grained tools that provide the more nuanced classification of the relative healthiness of QSR menu items may be needed, along with guidance for applying these tools in practice.

This study did not take into account price promotions that are tailored to an individual (e.g. through loyalty schemes) and/or applied on food delivery platforms (e.g. UberEats and Deliveroo) that are increasingly used for fast food^(54,55)^. Future studies should explore ways to capture personalised price promotions and price promotions on food delivery platforms. Finally, this study was not designed to explore the impact of price promotions on consumer purchases and population diets. This should be the subject of future studies, including consideration of the ways in which price promotions interact with other promotional techniques and pricing strategies (e.g. range of price points, menu structure) to influence consumer choices.

## Conclusion

Fast-food consumption is an important component of population diets in Australia. The current price promotion strategies adopted by major QSR chains in Australia are likely to encourage high levels of energy consumption and thereby contribute to unhealthy diets and obesity. Policies that monitor and address the strategies used by the fast-food industry to encourage consumption of unhealthy fast food need to be actively considered by governments. Specifically, policies to restrict price promotions on unhealthy foods and ensure lower-energy default items as part of combination deals should be considered as part of efforts to improve population diets and address obesity in Australia.

## Supplementary material

For supplementary material accompanying this paper visit https://doi.org/10.1017/S1368980021002688. click here to view supplementary material
