# Supplementary material for: Price promotions offered by quick service restaurants in Australia: analysis from an obesity prevention perspective
Source: Public Health Nutr. 2021 Jun 21;25(3):513–27. doi: 10.1017/S1368980021002688 (PMC9991702; doi:10.1017/S1368980021002688)
Supplement: Supplementary file 1 [file S1368980021002688sup001.docx]

**SUPPLEMENTARY MATERIAL**

**Supplementary Table 1:** Temporary price promotion (by product type) offered by ten major QSR chains in Australia between 25 November 2019 and 25 February 2020 (13 weeks)

| **QSR chain** | **Product type** | **Number of unique temporary price promotions identified** | **Mean promotion price**  **(AU $)**  *(95% CI)* | **Mean price reduction of promoted menu item(s) relative to regular price (AU$)**  *(95% CI)* | **Mean percentage (%) price reduction of promoted menu item(s) relative to regular price**  *(95% CI)* | **Mean energy content (kJ) of promoted menu item(s)**  *(95% CI)* | **Mean contribution of promoted menu item(s) to average daily recommended energy intake^1^ (%)**  *(95% CI)* | **Mean energy cost (cents per 100kJ)**  *(95% CI)* | **Classification as per Healthy Choices Framework** |
| --- | --- | --- | --- | --- | --- | --- | --- | --- | --- |
| McDonald’s | Main meal items only | 25 | 4.37  (3.48, 5.25) | 4.24  (3.43, 5.05) | 49.2  (45.69, 52.8) | 2790  (2234, 3346) | 32.1  (25.7, 38.5) | 16  (14, 19) | 100% Red |
|  | Combination deals for one person | 9 | 7.49  (4.41, 10.57) | 4.86  (2.22, 7.49) | 40.7  (21.9, 59.5) | 2991  (1731, 4252) | 34.4  (19.9, 48.9) | 14  (10, 19) | 100% Red |
|  | Combination deals for sharing | 1 | 24.95  (--) | 13.75  (--) | 35.5  (--) | 9969  (--) | NA | 25  (--) | 100% Red |
|  | Sides and/or drinks | 10 | 4.01  (2.17, 5.85) | 3.29  (2.17, 4.41) | 48.51  (41.67, 55.35) | 1974  (1096, 2852) | 22.7  (12.6, 32.8) | 23  (13, 32) | 80% Red, 20% Green^2^ |
|  | Desserts | 6 | 2.41  (0.54, 4.28) | 2.90  (2.46, 3.34) | 58.2  (45.8, 70.7) | 1829  (1461, 2197) | 21.0  (16.8, 25.3) | 12  (5, 19) | 100% Red |
| Hungry Jack’s | Main meal items only | 15 | 8.79  (7.53, 10.05) | 5.09  (4.34, 5.83) | 37.0  (33.9, 40.0) | 4745  (4050, 5440) | 54.5  (46.5, 62.5) | 19  (16, 23) | 100% Red |
|  | Combination deals for one person | 7 | 5.00  (4.08, 5.92) | 3.87  (3.07, 4.67) | 43.6  (37.6, 49.6) | 3526  (2871, 4182) | 40.5  (33.0, 48.1) | 15  (11, 18) | 100% Red |
|  | Combination deals for sharing | 10 | 15.34  (11.12, 19.55) | 10.99  (7.24, 14.74) | 41.4  (37.0, 45.8) | 9190  (6906, 11474) | NA | 16  (15, 18) | 100% Red |
|  | Sides and/or drinks | 3 | 2.33  (0.90, 3.77) | 1.91  (1.34, 2.48) | 45.3  (38.0, 52.6) | 845  (411, 1280) | 9.7  (4.7, 14.7) | 28  (23, 32) | 66.7% Red, 33.3% Green^2^ |
|  | Desserts | 3 | 2  (--) | 2.15  (1.49, 2.81) | 51.7  (44.3, 59.1) | 1873  (1300, 2445) | 21.5  (14.9, 28.1) | 11  (7, 14) | 100% Red |
| Domino’s | Main meal items only | 14 | 7.48  (5.74, 9.21) | 6.19  (4.09, 8.28) | 39.4  (31.8, 47.0) | 5317  (4030, 6604) | 61.1  (46.3, 75.9) | 16  (11, 21) | 100% Red |
|  | Combination deals for one person | - | - | - | - | - | - | - | - |
|  | Combination deals for sharing | 10 | 24.36  (18.10, 30.62) | 20.10  (14.68, 25.51) | 44.8  (41.5, 48.0) | 16432  (12539, 20326) | NA | 15  (13, 16) | 100% Red |
|  | Sides and/or drinks | 10 | 6.23  (4.50, 7.96) | 6.57  (2.45, 10.68) | 46.1  (34.4, 57.8) | 5227  (3610, 6844) | 60.1  (41.5, 78.7) | 12  (9, 15) | 100% Red |
|  | Desserts | - | - | - | - | - | - | - | - |
| Pizza Hut | Main meal items only | 5 | 22.53  (11.51, 33.55) | 10.59  (9.09, 12.09) | 35.9  (19.1, 52.7) | 15132  (8760, 21504) | 173.9  (100.7, 247.2) | 17  (3, 32) | 100% Red |
|  | Combination deals for one person | - | - | - | - | - | - | - | - |
|  | Combination deals for sharing | 16 | 32.20  (28.58, 35.82) | 6.92  (3.93, 9.91) | 16.4  (10.7, 22.1) | 22773  (19124, 26421) | NA | 15  (13, 17) | 100% Red |
|  | Sides and/or drinks | - | - | - | - | - | - | - | - |
|  | Desserts | 2 | 19.95  (--) | 0.95  (--) | 4.6  (--) | 7400  (--) | 85.1  (--) | 27  (--) | 100% Red |
| KFC | Main meal items only | 4 | 8.00  (1.64, 14.36) | 7.81  (-0.12, 15.74) | 48.4  (35.0, 61.8) | 4773  (521, 9025) | 54.9  (6.0, 103.7) | 17  (7, 27) | 100% Red |
|  | Combination deals for one person | 5 | 7.56  (3.52, 11.60) | 6.58  (1.79, 11.37) | 44.2  (27.9, 60.6 | 3407  (2040, 4775) | 39.2  (23.4, 54.9) | 22  (16, 28) | 100% Red |
|  | Combination deals for sharing | 9 | 21.17  (16.79, 25.55) | 14.58  (10.23, 18.94) | 40.1  (33.0, 47.3) | 11527  (8678, 14375) | NA | 19  (16, 22) | 100% Red |
|  | Sides and/or drinks | 4 | 3.90  (-1.54, 9.34) | 3.73  (0.86, 6.59) | 55.7  (22.0, 89.5) | 2522  (895, 4149) | 29.0  (10.3, 47.7) | 14  (-2, 29) | 100% Red |
|  | Desserts | - | - | - | - | - | - | - | - |
| Red Rooster | Main meal items only | 8 | 11.56  (7.39, 15.73) | 9.36  (5.64, 13.08) | 45.3  (34.2, 56.3) | 6425  (2716, 10134) | 73.8  (31.2, 116.5) | 21  (14, 28) | 100% Red |
|  | Combination deals for one person | 2 | 8.00  (--) | 11.49  (-18.50, 41.48) | 58.3  (-5.8, 122.4) | 5918  (-1262, 13098) | 68.0  (-14.5, 150.6) | 14  (-2, 30) | 100% Red |
|  | Combination deals for sharing | 8 | 24.54  (17.37, 31.71) | 18.72  (13.61, 23.83) | 43.7  (35.3, 52.1) | 15155  (11098, 19212) | NA | 16  (14, 18) | 100% Red |
|  | Sides and/or drinks | 3 | 4.67  (-4.06, 13.39) | 2.42  (1.15, 3.69) | 41.9  (-29.6, 113.4) | 3905  (-3820, 11629) | 44.9  (-43.9, 133.7) | 12  (9, 15) | 100% Red |
|  | Desserts | - | - | - | - | - | - | - | - |
| Subway | Main meal items only | 2 | 6.48  (-0.20, 13.15) | 1.78  (-0.45, 4.00) | 21.5  (17.7, 25.3) | 1581  (44, 3118) | 18.2  (0.5, 35.8) | 41  (-41, 124) | 100% Amber |
|  | Combination deals for one person | 1 | 8.00  (--) | 3.15  (--) | 28.3  (--) | 1513  (--) | 17.4  (--) | 53  (--) | 100% Amber |
|  | Combination deals for sharing | - | - | - | - | - | - | - | - |
|  | Sides and/or drinks | - | - | - | - | - | - | - | - |
|  | Desserts | - | - | - | - | - | - | - | - |
| Oporto | Main meal items only | 1 | 9.25  (--) | 9.25  (--) | 50  (--) | 5400  (--) | 62.1  (--) | 17  (--) | 100% Red |
|  | Combination deals for one person | - | - | - | - | - | - | - | - |
|  | Combination deals for sharing | - | - | - | - | - | - | - | - |
|  | Sides and/or drinks | - | - | - | - | - | - | - | - |
|  | Desserts | 1 | 11.5  (--) | 5  (--) | 30.30  (--) | 4780  (--) | 54.94  (--) | 24  (--) | 100% Red |
| Nando’s | Main meal items only | - | - | - | - | - | - | - |  |
|  | Combination deals for one person | 1 | 11  (--) | 4.85  (--) | 30.6  (--) | 4216  (--) | 48.6  (--) | 26  (--) | 100% Red |
|  | Combination deals for sharing | - | - | - | - | - | - | - | - |
|  | Sides and/or drinks | - | - | - | - | - | - | - | - |
|  | Desserts | - | - | - | - | - | - | - | - |
| Grill’d | Main meal items only | 1 | 15.08  (--) | 15.08  (--) | 50  (--) | 6827  (--) | 78.5  (--) | 22  (--) | 100% Red |
|  | Combination deals for one person | - | - | - | - | - | - | - | - |
|  | Combination deals for sharing | - | - | - | - | - | - | - | - |
|  | Sides and/or drinks | - | - | - | - | - | - | - | - |
|  | Desserts | - | - | - | - | - | - | - | - |

QSR: quick service restaurant

NA: not applicable

^1^ The average daily recommended energy intake for an adult is 8,700kJ.

^2^ Only drinks without added sugar (*e.g.*, coffee without added sugar).

**Supplementary Table 2:** Number and proportion of combination deals available on ‘regular menus’ of selected major QSR chains in Australia exceeding 30% of an adult’s average recommended daily energy intake (February 2020)

| **QSR chain^1^** | **Meal size** ^2^ | **Number of combination deals** | **Number of combination deals exceeding 30% of an adult’s average recommended daily energy intake ^3^** | | | |
| --- | --- | --- | --- | --- | --- | --- |
|  |  |  | **Lowest-energy option** | **Percentage**  **(%)** | **Highest-energy option** | **Percentage**  **(%)** |
| Hungry Jack’s | Small | 57 | 54 | 94.74 | 57 | 100.00 |
|  | Medium | 57 | 56 | 98.25 | 57 | 100.00 |
|  | Large | 57 | 57 | 100.00 | 57 | 100.00 |
| McDonald’s | Small | 46 | 13 | 28.26 | 41 | 89.13 |
|  | Medium | 46 | 13 | 28.26 | 46 | 100.00 |
|  | Large | 46 | 13 | 28.26 | 46 | 100.00 |
| KFC | Medium | 49 | 26 | 53.06 | 49 | 100.00 |
|  | Large | 48 | 26 | 54.17 | 48 | 100.00 |
| Subway | Medium | 30 | 28 | 93.33 | 30 | 100.00 |
|  | Large | 22 | 21 | 95.45 | 22 | 100.00 |
| Red Rooster | Small | 3 | 2 | 66.67 | 3 | 100.00 |
|  | Medium | 19 | 19 | 100.00 | 19 | 100.00 |
| Oporto | Medium | 12 | 12 | 100.00 | 12 | 100.00 |
| **Total** | | **492** | **340** | **69.11** | **487** | **98.98** |

QSR: quick service restaurant

^1^ Domino’s, Grill’d, and Domino’s did not offer combinational deals on their ‘regular menu’

^2^ Shared-sized combination deals not included

^3^ 30% of 8,700kJ = 2,610kJ

**Supplementary Table 3:** Mean energy cost of combination deals and incremental change in energy cost of combination deals (compared with relevant standalone main meal items) available on ‘regular menus’ of ten major QSR chains in Australia (February 2020)

| **QSR chain^1^** | **Meal size** | **Number of combination deals** | **Mean energy cost for standalone main meal item(s) (cents per 100kJ)** *(95% CI)* | **Mean energy cost for combination deals (cents per 100kJ)** | | **Incremental change in energy cost (combination deal compared with relevant standalone main meal item)**  **(cents/100kJ)** | |
| --- | --- | --- | --- | --- | --- | --- | --- |
|  |  |  |  | **Mean – Lowest-energy option**  *(95% CI)* | **Mean – Highest-energy option**  *(95% CI)* | **Mean – Lowest-energy option**  *(95% CI)* | **Mean – Highest -energy option**  *(95% CI)* |
| Hungry Jack’s  (n = 179) | Small | 57 | 26  (24, 28) | 27  (26, 29) | 23  (22, 24) | 31  (29, 33) | 18  (17, 19) |
|  | Medium | 57 | 26  (24, 28) | 28  (27, 30) | 23  (22, 24) | 33  (31, 35) | 19  (18, 20) |
|  | Large | 57 | 26  (24, 28) | 27  (26, 28) | 21  (20, 22) | 29  (27, 31) | 17  (16, 18) |
|  | Shared^2^ | 8 | 25  (22, 28) | 21  (18, 25) | 19  (17, 22) | 0  (-1, 1) | 13  (4, 22) |
| McDonald’s  (n = 138) | Small | 46 | 35  (31, 39) | 49  (44, 54) | 30  (28, 32) | 403  (383, 423) | 24  (23, 25) |
|  | Medium | 46 | 35  (31, 39) | 52  (47, 57) | 27  (26, 29) | 494  (481, 508) | 20  (20, 21) |
|  | Large | 46 | 35  (31, 39) | 56  (50, 61) | 25  (24, 26) | 579  (566, 593) | 18  (17, 18) |
| KFC  (n = 103) | Medium | 49 | 33  (31, 35) | 39  (37, 41) | 23  (22, 24) | 81  (73, 90) | 13  (12, 14) |
|  | Large | 48 | 33  (31, 35) | 48  (45, 51) | 20  (20, 21) | 151  (135, 166) | 13  (12, 14) |
|  | Shared^2^ | 6 | NA | 24  (21, 27) | 15  (13, 17) | NA | NA |
| Subway  (n = 52) | Medium | 30 | 62  (47, 77) | 42  (38, 45) | 31  (29, 33) | 30  (30, 30) | 18  (18, 18) |
|  | Large | 22 | 48  (36, 60) | 39  (34, 43) | 31  (28, 33) | 30  (30, 30) | 18  (18, 18) |
| Red Rooster  (n = 27) | Small | 3 | 41  (29, 53) | 27  (9, 45) | 23  (14, 32) | 6  (-22, 34) | 3  (-16, 23) |
|  | Medium | 19 | 46  (39, 54) | 32  (28, 35) | 26  (24, 29) | 12  (7, 18) | 8  (5, 12) |
|  | Large | 5 | 38  (28, 48) | 29  (26, 32) | 24  (20, 28) | 23  (20, 26) | 18  (16, 20) |
| Oporto  (n = 16) | Medium | 12 | 37  (33, 41) | 32  (31, 33) | 27  (25, 29) | 25  (19, 31) | 17  (12, 22) |
|  | Shared^2^ | 4 | 32  (29, 35) | 27  (21, 30) | 20  (12, 27) | 17  (8, 26) | 12  (5, 19) |

QSR: quick service restaurant

NA: not applicable

^1^ Domino’s, Grill’d, and Domino’s did not offer combinational deals on their ‘regular menu’. Energy costs could not be calculated for Pizza Hut as the main items in the combination deals were not fixed (*i.e.*, a selection of pizzas were offered)

^2^ Shared refers to combination deals that were clearly designed for sharing (*e.g.*, included more than one ‘main meal’ item as part of the deal, or were labelled as a ‘family’ size or ‘for sharing.

**Supplementary Table 4:** Number and proportion of combination deals available on ‘children’s menus’ of selected major QSR chains in Australia exceeding 30% of an 8-year-old child’s average recommended daily energy intake (February 2020)

| **QSR chain^1^** | **Number of combination deals** | **Number of combination deals exceeding 30% of an 8-year-old child’s average recommended daily energy intake ^2^** | | | |
| --- | --- | --- | --- | --- | --- |
|  |  | **Lowest-energy option** | **Percentage**  **(%)** | **Highest-energy option** | **Percentage**  **(%)** |
| McDonald’s | 7 | 0 | 0 | 7 | 100.00 |
| Grill’d | 6 | 2 | 33.33 | 2 | 33.33 |
| Hungry Jack’s | 4 | 0 | 0 | 4 | 100.00 |
| Red Rooster | 4 | 0 | 0 | 2 | 50.00 |
| Subway | 4 | 0 | 0 | 0 | 0 |
| Oporto | 4 | 3 | 75.00 | 3 | 75.00 |
| Nando’s | 3 | 0 | 0 | 1 | 33.33 |
| KFC ^3^ | 2 | 0 | 0 | 0 | 0 |
| **Total** | **34** | **5** | **14.71** | **19** | **55.88** |

QSR: quick service restaurant

^1^ Pizza Hut and Domino’s did not offer any children’s combination deals.

^2^ 30% of 7,100kJ = 2,130kJ

^3^ KFC offered a large-sized meal for their two children’s combination deals. For this analysis, the ‘large-sized’ versions of these combination deals were excluded from analysis, as KFC was the only QSR chain that offered a large size.

**Supplementary Table 5:** Price savings, energy content and healthiness of combination deals on the ‘breakfast menus’ of selected major QSR chains in Australia (February 2020)

| **QSR chain^1^** | **Size of meals** | **Number of meals** | **Price saving per combination deal, relative to price of individual items** | | | | **Mean energy cost for standalone main meal items (cents per 100kJ)**  *(95% CI)* | **Mean energy cost for combination deals (cents per 100kJ)** | | **Incremental change in energy cost (combination deal compared with relevant standalone main meal item)**  **(cents/100kJ)** | | **Energy content per combination deal** | | | | **Classification as per Healthy Choices Framework** |
| --- | --- | --- | --- | --- | --- | --- | --- | --- | --- | --- | --- | --- | --- | --- | --- | --- |
|  |  |  | **Mean reduction ($) – lowest option**  *(95% CI)* | **Mean reduction (% of price of individual items) – lowest option**  *(95% CI)* | **Mean reduction ($) – highest option**  *(95% CI)* | **Mean reduction (% of price of individual items) – highest option**  *(95% CI)* |  | **Mean – Lowest-energy option**  *(95% CI)* | **Mean – Highest-energy option**  *(95% CI)* | **Mean – Lowest-energy option**  *(95% CI)* | **Mean – Highest energy option**  *(95% CI)* | **Mean energy content (kJ) – lowest-energy option**  *(95% CI)* | **Mean contribution to average daily recommended energy intake^2^ – lowest-energy option (%)**  *(95% CI)* | **Mean energy content (kJ) – highest-energy option**  *(95% CI)* | **Mean contribution to average daily recommended energy intake^2^– highest-energy option (%)**  *(95% CI)* |  |
| McDonald’s | Medium | 8 | 0.37  (0.22, 0.52) | 3.7  (2.4, 5.1) | 1.57  (1.42, 1.72) | 14.4  (13.3, 15.4) | 30  (26, 34) | 41  (35, 46) | 26  (24, 28) | 72  (69, 75) | 23  (22, 24) | 2383  (1823, 2943) | 27.4  (21.0, 33.8) | 3653  (3093, 4213) | 42.0  (35.6, 48.4) | 100% Red |
| Hungry Jack’s | Small | 19 | 2.58  (1.89, 3.27) | 22.5  (18.3, 26.8) | 3.53  (2.84, 4.22) | 28.9  (25.2, 32.7) | 27  (25, 29) | 31  (29, 33) | 23  (22, 25) | 40  (35, 45) | 20  (18, 21) | 2823  (2506, 3140) | 32.4  (28.8, 36.1) | 3653  (3336, 3970) | 42.00  (38.3, 45.6) | 100% Red |
|  | Medium | 19 | 2.59  (1.91, 3.28) | 21.7  (17.6, 25.8) | 3.04  (2.36, 3.73) | 24.8  (20.9, 28.6) | 27  (25, 29) | 32  (30, 35) | 21  (20, 22) | 46  (41, 52) | 17  (16, 17) | 2823  (2506, 3140) | 32.4  (28.8, 36.1) | 4233  (3916, 4550) | 48.7  (45.0, 52.3) | 100% Red |
|  | Large | 19 | 2.09  (1.41, 2.78) | 17.2  (12.9, 21.5) | 2.94  (2.26, 3.63) | 23.1  (19.4, 26.9) | 27  (25, 29) | 34  (32, 37) | 21  (20, 22) | 53  (46, 59) | 17  (16, 18) | 2823  (2506, 3140) | 32.4  (28.8, 36.1) | 4479  (4179, 4780) | 51.5  (48.0, 54.9) | 100% Red |
| Subway | Medium | 8 | 1.75  (1.75, 1.75) | 13.3  (12.4, 14.2) | 1.75  (1.75, 1.75) | 13.3  (12.4, 14.2) | 36  (29, 44) | 38  (32, 44) | 27  (25, 30) | 45  (45, 45) | 20  (20, 20) | 3141  (2432, 3850) | 36.1  (28.0, 44.3) | 4254  (3545, 4963) | 48.9  (40.8, 57.0) | 100% Red |
|  | Large | 4 | 1.75  (--) | 11.3  (10.6, 12.0) | 1.75  (--) | 11.3  (10.6, 12.0) | 33  (30, 36) | 36  (33, 38) | 28  (26, 29) | 45  (45, 45) | 20  (20, 20) | 3885  (3350, 4420) | 44.7  (38.5, 50.8) | 4998  (4463, 5533) | 57.4  (51.3, 63.6) | 100% Red |
| QSR: quick service restaurant  ^1^ KFC, Red Rooster, Pizza Hut, Oporto, Domino’s, Grill’d, and Nando’s did not offer ‘breakfast only’ combination deals.  ^2^ The average daily recommended energy intake for an adult is 8,700kJ. | | | | | | | | | | | | | | | | |

**Supplementary Table 6:** Mean energy cost of combination deals and incremental change in energy cost of combination deals (compared with relevant standalone main meal items) available on ‘children’s menus’ of selected major QSR chains in Australia (February 2020)

| **QSR chain^1^** | **Number of combination deals** | **Mean energy cost of standalone main meal item(s) (cents per 100kJ)** *(95% CI)* | **Mean energy cost for combination deals (cents per 100kJ)** | | **Incremental change in energy cost (combination deal compared with relevant standalone main meal item)**  **(cents/100kJ)** | |
| --- | --- | --- | --- | --- | --- | --- |
|  |  |  | **Mean – Lowest-energy option**  *(95% CI)* | **Mean – Highest-energy option**  *(95% CI)* | **Mean – Lowest-energy option**  *(95% CI)* | **Mean – Highest energy option**  *(95% CI)* |
| McDonald’s | 7 | 38  (20, 57) | 73  (39, 107) | 19  (15, 24) | 430  (276, 584) | 11  (7, 15) |
| Hungry Jack’s | 4 | 35  (9, 62) | 53  (30, 76) | 21  (17, 25) | 215  (79, 350) | 14  (9, 20) |
| KFC^2^ | 2 | 31  (-80, 142) | 41  (21, 62) | 17  (14, 21) | 78  (-412, 568) | 10  (-54, 74) |
| Red Rooster | 4 | 39  (24, 54) | 52  (21, 84) | 28  (19, 37) | 53  (-132, 237) | 11  (-27, 48) |
| **Total** | **34^3^** | **38**  **(32, 43)** | **57**  **(49, 65)** | **36**  **(29, 43)** | **250**  **(143, 357)** | **12**  **(8, 16)** |
| QSR: quick service restaurant  ^1^ Pizza Hut and Domino’s did not offer any children’s combination deals.  ^2^ KFC offered a large-sized meal for their two children’s combination deals. For this analysis, the ‘large-sized’ versions of these combination deals were excluded from analysis, as KFC was the only QSR chain that offered a large size.  ^3^ Nando’s (n=3), Grill’d (n=6), Subway (n=4), and Oporto (n=4) offered children’s combination deals. However, these QSR chains did not offer the main meal items in the children’s combination deals as individual or *ala carte* items; hence, the energy costs could not be calculated as individual pricing for the main meal items were unavailable. | | | | | | |
